# Influence of atorvastatin on coronary calcifications and myocardial perfusion defects in systemic lupus erythematosus patients: a prospective, randomized, double-masked, placebo-controlled study

**DOI:** 10.1186/ar3402

**Published:** 2011-07-20

**Authors:** Wojciech Plazak, Krzysztof Gryga, Hanna Dziedzic, Lidia Tomkiewicz-Pajak, Malgorzata Konieczynska, Piotr Podolec, Jacek Musial

**Affiliations:** 1 Department of Cardiac and Vascular Diseases, the John Paul II Hospital, Jagiellonian University Medical College, Pradnicka Str 80, 31-202 Krakow, Poland; 2 Department of Internal Medicine, Jagiellonian University Medical College, Skawinska Str 8, 31-066 Krakow, Poland; 3 Center for Diagnosis, Prevention and Telemedicine, the John Paul II Hospital, Jagiellonian University Medical College, Pradnicka Str 80, 31-202 Krakow, Poland

**Keywords:** systemic lupus erythematosus, autoimmune diseases, coronary calcification, accelerated atherosclerosis, MDCT, perfusion scintigraphy, statins

## Abstract

**Introduction:**

Mortality in systemic lupus erythematosus (SLE) patients is influenced by an increased occurrence of severe cardiovascular complications. Statins have been proven to protect a wide spectrum of SLE patients from these complications. This study was conducted to determine the possible efficacy of atorvastatin in SLE patients as assessed by multi-detector computed tomography (MDCT)-based coronary calcium scoring and single photon emission computed tomography (SPECT) of the myocardium.

**Methods:**

Sixty SLE patients in stable clinical conditions were randomized to receive either atorvastatin (40 mg daily; *n *= 28) or placebo (*n *= 32). Clinical and biochemical evaluation together with MDCT-based coronary calcium scoring and SPECT studies (Tc-99 m sestamibi) were performed at the time of randomization and after 1 year of treatment.

**Results:**

At randomization, SPECT revealed perfusion defects at rest in 22 (36.7%) patients and exercise-induced defects in 8 (13.3%), whereas MDCT revealed coronary calcifications in 15 subjects (25%). Coronary calcium deposits increased after 1 year in the placebo group (plaque volume change from 35.2 ± 44.9 to 62.9 ± 72.4, *P *< 0.05; calcium score from 32.1 ± 39.1 to 59.5 ± 64.4; *P *< 0.05), but not in the atorvastatin group (plaque volume 54.5 ± 62.4 vs. 51.0 ± 47.6, *P *not significant; calcium score 44.8 ± 50.6 vs. 54.9 ± 62.5, *P *not significant). The atorvastatin group showed a decrease in total serum cholesterol (from 5.1 ± 1.2 to 4.4 ± 0.7 mmol/L, *P *< 0.05), LDL cholesterol (2.9 ± 1.0 to 2.3 ± 0.6 mmol/L, *P *< 0.05), triglycerides (1.6 ± 0.6 to 1.2 ± 0.5 mmol/L, *P *< 0.05), and C-reactive protein (CRP) (4.4 ± 4.1 to 2.7 ± 1.7 mg/L, *P *< 0.05). There was no change in the mean Systemic Lupus Erythematosus Disease Activity Index (SLEDAI) score in patients from both groups. Perfusion defects observed at randomization showed no change after one year treatment with atorvastatin.

**Conclusions:**

In SLE patients 40 mg of atorvastatin daily for 1 year led to a decrease in serum lipids and CRP levels. Additionally the progression of atherosclerosis, as assessed by MDCT-based coronary calcium scoring, is restrained by atorvastatin treatment. The value of statin treatment in patients with SLE free from cardiovascular disease clinical symptoms should be addressed in large, prospective clinical trials.

## Introduction

Systemic lupus erythematosus (SLE) is a generalized autoimmune disease, in which diffuse, chronic inflammatory reactions play an important pathogenic role. Contemporary mortality of SLE patients is mainly due to severe cardiovascular complications [1]. Suggested factors that may influence accelerated arteriosclerosis include a generalized, chronic inflammation and corticosteroid usage [2]. The relation between increased levels of inflammatory cytokines and life-threatening cardiovascular episodes has been well-documented [3]. However, the optimal strategy for the prevention of atherosclerosis in SLE patients is not established.

Statins, HMG-CoA reductase inhibitors, are widely used in the treatment of hyperlipidemia and prevention against cardiovascular disease. In the general population, large randomized controlled trials have demonstrated their beneficial effects in hypercholesterolemia treatment [4], as well as primary and secondary prevention of coronary artery disease [5-7] with the regression of established coronary atherosclerosis [8]. Interestingly, the magnitude of the protection and decrease in mortality afforded by statins cannot be explained entirely by their cholesterol-lowering effect. It has been shown, among others, that statins exert strong anti-inflammatory action [9] and ameliorate endothelial dysfunction, protecting from inflammation-induced endothelial injury [10,11].

Statins are recommended for patients with SLE at high cardiovascular risk with diagnosed coronary artery disease, but these recommendations are based on the extrapolation of the results obtained in non-SLE populations [12-16]. There has been little evidence for the effectiveness of statins in cardiovascular symptom-free SLE patients. Implementation of multi-detector computed tomography (MDCT) and single photon emission computerized tomography (SPECT) allows for a non-invasive evaluation of coronary atherosclerosis and myocardial perfusion abnormalities, and enables the assessment of statin influence on coronary artery structural changes and heart function.

This study was conducted to determine the effect of atorvastatin treatment on MDCT-based coronary calcium scoring and SPECT-assessed myocardial perfusion abnormalities in SLE patients free of clinical symptoms of cardiovascular disease.

## Materials and methods

The study was performed in 60 consecutive patients treated for systemic SLE in the Department of Internal Medicine, Jagiellonian University Medical College, Krakow. All patients fulfilled at least four American College of Rheumatology classification criteria for SLE [17,18] and were in stable clinical conditions (no need for immunosuppressive therapy intensification, i.e. current immunosuppressive drug dose increase or introduction of an additional immunosuppressive drug within the past three months). Patients with known cancer, clinical symptoms of coronary heart disease or heart failure (New York Heart Association III or IV class), renal failure (creatinine clearance < 30 ml/min), and/or respiratory failure were excluded from the study.

Atorvastatin was chosen for this study because of its superiority over two other statins (simvastatin and pravastatin) in the inhibition of atherosclerosis shown by two large clinical trials [19,20]. We chose, however, a daily dose of 40 mg to limit treatment-associated adverse events.

Patients were randomized (random option in Microsoft Excel software, Qumak Secom SA, Warsaw, Poland) to atorvastatin (40 mg, in the evening) or placebo group. Placebo group received shape and color-matched placebo tablets at the same time. The duration of the study was one year. All parameters described below were assessed at randomization and after one year of treatment by medical staff, unaware of the type of treatment.

The SPECT study (ECAM Gamma Camera, Siemens, Munich, Germany) was performed at rest and during exercise in a two-day protocol. At the first day, at near maximal stress, a 25 to 40 mCi dose of Tc-99 m sestamibi was injected (actual patient dose was modified taking into account patients weight) and exercise continued for one additional minute after injection. Tc-99 m sestamibi SPECT imaging was begun 15 to 30 minutes later. On the second day rest examinations were performed. SPECT was performed using a circular 180° acquisition for 60 projections at 20 seconds per projection. Myocardial perfusion was assessed in 17 left ventricle myocardial segments. The number of segments with persistent or exercise-induced perfusion defects were assessed by visual interpretation.

Coronary calcium scoring was performed using a multidetector CT imager (Somatom Definition, Siemens, Munich, Germany). The images were ECG triggered with 3 mm thick sections obtained covering the whole heart. Coronary artery calcifications were defined as lesions with attenuation greater than 130 HU in more than four adjacent pixels. For the quantification of coronary calcium 3D Leonardo application (Siemens, Munich, Germany) was used. The number of atherosclerotic plaques in particular coronary arteries and its volume were assessed. The Agatson calcium score was calculated [21].

Laboratory tests included determination of serum antinuclear antibodies (ANA) presence, their titer (indirect immunofluorescence; Hep-2 cells; Euroimmun GmbH, Lubeck, Germany) and type (immunoblotting; Euroline System, Euroimmun GmbH, Lubeck, Germany), serum concentrations of C-reactive protein (CRP), and complement C3c and C4 components by nephelometry (Siemens, Munich, Germany).

In addition, serum levels of anticardiolipin (aCL) and antiβ2GPI antibodies (of both, IgG and IgM class) were measured using home-made ELISA with the Sapporo standard for antiβ2GPI antibody measurements (HCAL for IgG, EY2C9 for IgM), as previously described [22]. The values exceeding 99^th ^percentile of a healthy population sample were considered positive.

Lupus anticoagulant (LA) was determined in accordance with the three-step procedure recommended by the International Society on Thrombosis and Haemostasis [23].

Statistical analysis was performed using Statistica Six Sigma software (StatSoft, Krakow, Poland). All numerical data were expressed as mean values ± standard deviations, as median values or as proportions. Continuous variables were compared using a t-test. Chi-square test was used to examine differences in proportions. The level for statistical significance was predetermined at *P *< 0.05.

Before the study, an informed consent was obtained from each patient. The study protocol conforms to the ethical guidelines of the 1975 Declaration of Helsinki. The study was approved by the Ethical Committee of the Jagiellonian University in Krakow, Poland.

## Results

The study group consisted of 54 (90%) females and 6 (10%) males, aged 20 to 73 years (mean 41.8 years). Twenty eight patients formed the atorvastatin group and 32 patients belonged to the placebo group. Three subjects were previously diagnosed with antiphospholipid syndrome (APS) based on the revised APS classification criteria [24]. One of these three suffered from an objectively confirmed pulmonary embolism. ECG recordings were normal in all the patients. Results of peripheral blood count, serum sodium, potassium, glucose, creatinine, and urinalysis were all normal. Systemic Lupus Erythematosus Disease Activity Index (SLEDAI) score [25] at randomization ranged from 0 to 20 (median 4). The main complaints at inclusion were arthralgias and main laboratory abnormalities - low complement levels and increased ANA titers (four patients were ANA negative; Table 1). Immunosuppressive treatment included: methylprednisolone in 32 (53.3%) subjects (≤ 4 mg for clinical stability maintenance), prednisone in 2 (3.3%), chloroquine derivate in 5 (8.3%), azathioprine in 4 (6.7%), cyclophosphamide in 3 (5%), and methotrexate in 2 (3.3%). The other 12 patients did not use any immunosuppressive drugs in the past 12 months of observation. Other treatments included angiotensin converting enzyme inhibitors in 4 (6.7%) subjects, beta blockers in 3 (5%) and calcium channel blockers in 2 (3.3%). APS patients were treated with anticoagulant (warfarin, two patients) or antiplatelet therapy (aspirin, one patient). The above described pharmacotheraphy remained unchanged during the one-year treatment period.

**Table 1:** Autoantibodies and other laboratory parameters in SLE patients at randomization

	Range (mean ± SD)	Number (%) of patients with out-of-range values
ANA (titer)	0-1/20480	56 (93.3%)
C3c (g/l)	0.43-1.39 (0.90 ± 0.25)	32 (53.3%)
C4 (g/l)	0.02-0.26 (0.13 ± 0.05)	16 (26.7%)
LA	-	11 (18.3%)
aCL IgG (RU/ml)	0.68-121.56 (14.4 ± 20.3)	20 (33.3%)
aCL IgM (RU/ml)	1.62-52.93 (12.1 ± 10.6)	26 (43.3%)
antiβ2GPI IgG (RU/ml)	0.16-95.33 (3.8 ± 15.3)	8 (13.3%)
antiβ2GPI IgM (RU/ml)	0.14-21.66 (2.2 ± 3.7)	24 (40%)

aCL, anticardiolipin antibodies; ANA, antinuclear antibodies; antiβ2GPI, antiβ2-glycoprotein I antibodies; LA, lupus anticoagulant; SD, standard deviation; SLE, systemic lupus erythematosus.

Abnormal low levels for C3c: < 0.9 g/l, for C4: < 0.1 g/l. Cut-off value for aCL IgG: > 20 RU/ml, aCL IgM: > 30 RU/ml, antiβ2GPI IgG: > 3 RU/ml, antiβ2GPI IgM > 2.6 RU/ml.

Baseline characteristics of the study patients by placebo/atorvastatin group is shown in Table 2.

**Table 2:** Baseline characteristics of the study patients by group

	Placebo group (*n *= 32)	Atorvastatin group (*n *= 28)	*P*
Age (years)	41.4 ± 12.4	41.8 ± 13.4	ns
Gender (females/males)	30/2	24/4	ns
Arterial hypertension (n (%))	2 (6.3%)	1 (3.6%)	ns
Diabetes mellitus (n (%))	0 (0%)	0 (0%)	ns
Obesity (n (%))	0 (0%)	0 (0%)	ns
Tobacco smoking (n (%))	1 (3.1%)	1 (3.6%)	ns
Total cholesterol (mmol/l)	4.5 ± 0.8	5.1 ± 1.2	ns
LDL cholesterol (mmol/l)	2.6 ± 0.8	2.9 ± 1.0	ns
HDL cholesterol (mmol/l)	1.4 ± 0.3	1.4 ± 0.3	ns
Triglycerides (mmol/l)	1.2 ± 0.5	1.6 ± 0.6	< 0.05
CRP (mg/l)	4.0 ± 8.9	4.4 ± 4.1	ns
Number of patients with plaques in MDCT (n (%))	9 (28.1%)	6 (21.4%)	ns
Plaque volume (mm^3^)	35.2 ± 44.9	54.5 ± 62.4	ns
Calcium score	32.1 ± 39.1	44.8 ± 50.6	ns
Number of patients with perfusion defects in SPECT	18 (56.3%)	12 (42.9%)	ns
Number of underperfused myocardial segments (median)	3 (9.4%)	3 (10.7%)	ns

CRP, C-reactive protein; HDL, high-density lipoprotein; LDL, low-density lipoprotein; MDCT, multi-detector computed tomography; ns, not significant; SPECT, single photon emission computed tomography.

During the entire observation period, pathologic results of SPECT or MDCT were found in 37 (61.6%) out of 60 patients examined.

At randomization, SPECT study revealed myocardial perfusion abnormalities in 30 (50.0%) patients, persistent defects in 22 (36.7%) patients, and exercise-induced defects in 8 (13.3%). The number of myocardial segments with persistent defects ranged from two to five (median three), and with exercise-induced defects from one to four (median three). Perfusion abnormalities were observed predominantly in the region supplied by the left anterior descending artery (22 patients, 73%), but also in the right coronary artery (three patients, 10%) or left anterior descending together with right or circumflex arteries (five patients, 17%). Out of 30 patients with perfusion abnormalities, in 21 (70%) the typical signs of ischemia (horizontal or down-slope ST depression ≥ 0.1 mV) were visible in ECG recordings during exercise.

At randomization, MDCT revealed coronary calcifications in 15 (25%) patients. The number of atherosclerotic calcified plaques ranged from 2 to 13 (median 3), its volume 4 to 156.4 mm^3 ^(mean 45.5 ± 58.6). Calcium scores ranged from 2 to 138.9 (mean 39.9 ± 50.9). Calcifications were present in left anterior descending artery (eight patients, 53%), right coronary artery (two patients, 13%), left anterior descending with right coronary artery (one patient, 7%) or all three arteries (four patients, 27%).

Of the group of patients with any pathology in SPECT or MDCT at baseline (*n *= 36, 100%), myocardial perfusion abnormalities accompanied by the presence of coronary calcifications were present in nine (25%) patients. In 21 (58%) patients, SPECT study was abnormal despite the lack of coronary calcifications (calcium score = 0). On the other hand, in six (17%) patients with mild calcium deposits (two to three plaques, calcium score 4.4 to 35.1 (mean 14.8 ± 14.2)) SPECT study did not show any perfusion defects.

**Figure 1:**
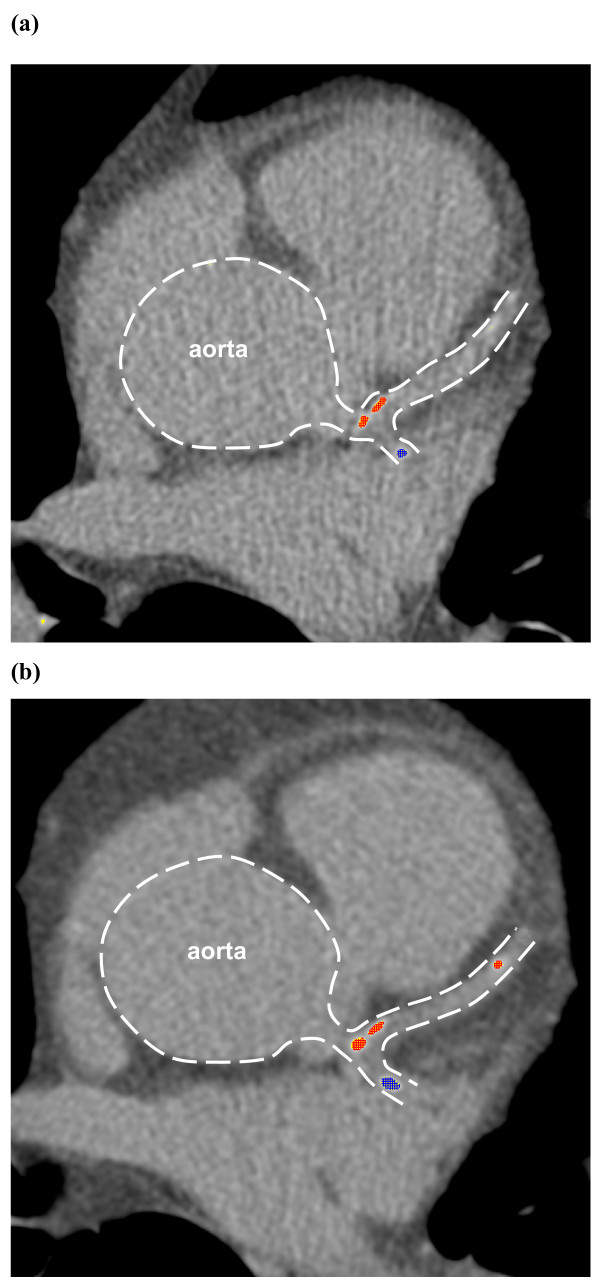
**The examples of multi-detector computed tomography in a patient from the (a) placebo group at randomization and (b) after one year**. **a) **At randomization, two calcified plaques are seen in left anterior descending artery (red colour) and one calcified plaque in circumflex artery (blue colour). Plaques volume 156.4 mm^3^, calcium score 138.9. **b) **After one year, the volume of previously observed plaques increased with the new calcification in distal part of left anterior descending artery. Plaques volume 223 mm^3^, calcium score 202.5.

During one-year observation progression of atherosclerosis was observed only in the placebo group (Table 3). Out of nine patients with coronary plaques at randomization, the increase of plaque volume (> 10 mm^3^) after one year was observed in five (55.6%). In one patient free of calcium deposits at randomization, new plaques appeared after one year. As a result, the mean coronary plaque volume and calcium score increased significantly (Table 3). An example of atherosclerosis progression in a patient from the placebo group is shown in Figure 1.

**Table 3:** Coronary calcium score, number and volume of coronary plaques in SLE patients from the placebo group and atorvastatin group at randomization and after one year of treatment

	At randomization	After one year	*P*
	
Placebo group, *n *= 32			
Number of patients with plaques	9 (28.1%)	10 (31.3%)	ns
Plaque volume (mm^3^)	35.2 ± 44.9	62.9 ± 72.4	< 0.05
Number of plaques	2-13 (median 4)	1-12 (median 5)	ns
Calcium score	32.1 ± 39.1	59.5 ± 54.4	< 0.05
Atorvastatin group, *n *= 28			

Number of patients with plaques	6 (21.4%)	6 (21.4%)	ns
Plaque volume (mm^3^)	54.5 ± 62.4	51.0 ± 47.6	ns
Number of plaques	2-4 (median 2)	1-8 (median 2)	ns
Calcium score	44.8 ± 50.6	54.9 ± 62.5	ns

ns, not significant; SLE, systemic lupus erythematosus.

In the atorvastatin group, there was no increase of plaque volume (> 10 mm^3^) in any of the six patients with deposits found at randomization. Also, the mean coronary plaque volume and calcium score did not change (Table 3).

The number of patients with perfusion defects and the number of myocardial segments with persistent or exercise-induced defects in the SPECT study remained unchanged during one-year observation in neither group of patients studied (Table 4).

**Table 4:** Persistent and exercise-induced myocardial perfusion defects in SLE patients from placebo group and atorvastatin group at randomization and after one year of treatment

	At randomization	After one year	*P*
	
Placebo group, *n *= 32			
Number of patients with persistent perfusion defects	14 (43.8%)	11 (34.3%)	ns
Number of persistently underperfused segments	2-5 (median 3)	3-6 (median 3)	ns
Number of patients with exercise-induced perfusion defects	4 (12.5%)	6 (18.8%)	ns
Number of underperfused myocardial segments at exercise	1-4 (median 3)	2-3 (median 3)	ns
Atorvastatin group, *n *= 28			
Number of patients with persistent perfusion defects	8 (28.6%)	8 (28.6%)	ns
Number of persistently underperfused segments	1-5 (median 3)	2-6 (median 3)	ns
Number of patients with exercise-induced perfusion defects	4 (14.3%)	5 (17.9%)	ns
Number of underperfused myocardial segments at exercise	2-4 (median 3)	3-6 (median 3)	ns

ns, not significant; SLE, systemic lupus erythematosus.

After one year of treatment, total serum cholesterol decreased promptly by 13%, low-density lipoprotein (LDL) cholesterol by 21%, triglycerides by 25% and CRP concentration by 39% in the atorvastatin group, but remained unchanged in the placebo group (Table 5). There was no change in the activity of alanine aminotransferase (ALT) and aspartate aminotransferase (AST) nor creatine phosphokinase (CPK) in either group, except for one patient in the placebo group (Table 5). There was no need for atorvastatin discontinuation in any of the patients.

**Table 5:** Biochemical data and SLEDAI score in SLE patients from atorvastatin group and from placebo group at randomization and after one year of treatment

	At randomization	After one year	*P*
	
Atorvastatin group, *n *= 28			
Total cholesterol (mmol/l)	5.1 ± 1.2	4.4 ± 0.7	< 0.05
LDL cholestrol (mmol/l)	2.9 ± 1.0	2.3 ± 0.6	< 0.05
HDL cholesterol (mmol/l)	1.4 ± 0.3	1.4 ± 0.3	ns
Triglycerides (mmol/l)	1.6 ± 0.6	1.2 ± 0.5	< 0.05
CRP (mg/l)	4.4 ± 4.1	2.7 ± 1.7	< 0.05
ALT (IU/l)	23.9 ± 6.7	22.4 ± 6.9	ns
AST (IU/l)	22.9 ± 3.7	31.5 ± 6.2	ns
CPK (IU/l)	70.0 ± 78.2	62.9 ± 47.2	ns
SLEDAI	2-20 (median 4)	0-20 (median 4)	ns
Placebo group, *n *= 32			

Total cholesterol (mmol/l)	4.5 ± 0.8	4.5 ± 0.7	ns
LDL cholestrol (mmol/l)	2.6 ± 0.8	2.6 ± 0.8	ns
HDL cholesterol (mmol/l)	1.4 ± 0.3	1.4 ± 0.3	ns
Triglycerides (mmol/l)	1.2 ± 0.5	1.3 ± 0.6	ns
CRP (mg/l)	4.0 ± 8.9	3.9 ± 5.1	ns
ALT (IU/l)	27.1 ± 8.6	39.1 ± 51.4*	ns
AST (IU/l)	26.1 ± 6.2	40.2 ± 56.6*	ns
CPK (IU/l)	53.2 ± 37.5	71.2 ± 57.2	ns
SLEDAI	0-12 (median 4)	0-12 (median 2)	ns

* in one patient increased ALT (248 IU/l) and AST (273 IU/l) levels were observed

CRP, C-reactive protein; ALT, alanine aminotransferase; AST, aspartate aminotransferase; CPK, creatine phosphokinase; HDL, high-density lipoprotein; LDL, low-density lipoprotein; ns, not significant; SLE, systemic lupus erythematosus; SLEDAI, Systemic Lupus Erythematosus Disease Activity Index.

Mean value of the SLEDAI score remained unchanged in both groups (Table 5). During the treatment period, SLE flare (SLEDAI increase ≥ 3) was observed in two patients from the atorvastatin group and in one from the placebo group. In two atorvastatin group patients, the SLEDAI increase (from 8 to 12 and from 4 to 8 points) resulted solely from the onset of hematuria. In one patient from the placebo group, SLEDAI increase (from 4 to 10 points) resulted from the onset of both hematuria and pyuria.

## Discussion

The major finding of this study is the inhibition of atherosclerosis progression by atorvastatin in SLE patients as evidenced by MDCT-based calcium scoring. To our knowledge, it is the first report showing such a beneficial effect of statin therapy in this population at high risk of life-threatening cardiovascular complications. The volume of coronary calcified plaques was stable in the active-treatment group, and increased significantly in the placebo group. At the same time, coronary calcium score increased significantly in the placebo group only.

Patients with SLE suffer from premature atherosclerosis. Our study supports previously published data on high frequency of myocardial perfusion defects in SLE patients as demonstrated by the SPECT study [26,27]. Perfusion defects were present in 50% of cases, despite normal ECG recordings at rest and lack of any clinical symptoms of myocardial ischemia. Predominantly persistent perfusion abnormalities were detected. In most of the patients, the number of underperfused left ventricle segments was low. However, it has been already established that the presence of even small perfusion defects in the SPECT study strongly affects prognosis [28,29]. Beside the presence of myocardial perfusion defects, 25% of our asymptomatic SLE patients showed calcified atherosclerotic changes in their coronary arteries. It is the most frequent localization of such changes in SLE, as shown in another study of 50 SLE patients, where the frequency of atherosclerotic plaques observed in MDCT were the highest in coronary arteries (42% of patients with calcifications), followed by carotid arteries (24% of patients with calcifications) [30]. A study of 157 SLE patients showed that in subjects with the mean age of 40 years - comparable with the age of our patients - the frequency of coronary artery calcifications is 30 to 40% [31]. This percentage is relatively higher than in the general population: in the study of 35,388 subjects calcium scores above 10 were observed in only 10% of cases, and calcium scores above 100 in 2% [32]. Coronary calcium deposits provide an independent indication of a short- and long-term risk of cardiac events, even in patients with normal SPECT results [33-35].

Our results support also the published data showing higher frequency of myocardial perfusion abnormalities detected by SPECT as compared with the frequency of coronary calcium deposits detected by MDCT in SLE population [26,27,31]. This might be partially explained by the fact that antiphospholipid antibodies are associated with thrombotic events in coronary beds, rather than with subclinical atherosclerosis [36]. Thrombosis in coronary arteries leads to perfusion defects detectable by SPECT, but not by MDCT. Calcified plaques may develop in time at the basis of thrombi or may form due to endothelium dysfunction. In the present study the patients with perfusion abnormalities despite the lack of coronary calcifications were observed. On the other hand, small coronary plaques may have no influence on the perfusion: the patients with normal perfusion despite small calcium deposits in the arteries were also observed.

The inhibition of atherosclerosis progression in SLE patients by atorvastatin seems of major importance for their prognosis. In a seven-year prospective follow-up study in a group of 1,126 otherwise healthy subjects, Chang et al. showed that the risk of myocardial infarction or the need for revascularization correlated with the patients calcium score and occurred at higher frequency in subjects with calcium score above 100 [33]. In our study, the mean value of the calcium score was lower (39.9 ± 50.9) and the follow-up period much shorter, but the significant progression of atherosclerosis in the placebo group (increase of mean calcium score by 85.4% during one year) may have important clinical implications for patients' future.

Atorvastatin did not influence myocardial perfusion as assessed by SPECT. Calcium deposits in coronary arteries revealed by MDCT were obviously too small to result in any significant persistent or exercise-induced perfusion defects.

It has been shown that statins exert not only anti-lipid, but also marked anti-inflammatory effects [9]. Accordingly, in our study serum concentrations of total cholesterol, LDL cholesterol, and triglycerides all decreased after atorvastatin treatment. Importantly, this was accompanied by the decrease in CRP despite an unchanged immunosuppressive therapy. It was previously shown that the magnitude of protection and the decrease in mortality afforded by statins cannot be entirely explained by their cholesterol-lowering effect [10]. A large study of 3,745 patients showed that patients who have low CRP levels after statin therapy have better clinical outcome than those with higher CRP levels, regardless of the resultant level of LDL cholesterol decrease [9]. The ability of atorvastatin to lower CRP concentrations shown in this study is of major importance for SLE patients, as an ongoing chronic inflammation presents as the major mechanism of systemic SLE complications.

Recently, the Lupus Atherosclerosis Prevention Study has been completed [37], based on the methodology similar to that described above. The authors found a greater increase in coronary artery calcium score in the placebo group, but due to a calcium score increase observed also in the atorvastatin group, the inter-group change was not statistically significant. There was, however, a significant difference in favor of atorvastatin in the proportion of patients in whom carotid intima-media thickness improved, stayed the same, or got worse. Surprisingly, during follow up, a greater decrease of CRP level was observed in the placebo group as compared with the atorvastatin group.

Statin therapy in SLE may be complicated by the reported cases of statin-induced lupus-like syndrome [38-40]. Pathogenic mechanisms may include increased cellular apoptosis induced by statins [41] and/or direct immunomodulatory effect of statins on T lymphocytes [42]. In our patients, no changes typical of any statin-related adverse events were observed. Liver enzyme and CPK levels were normal in all active-treated subjects. There was also no other adverse effects that would require discontinuation of therapy.

Our results may have important implications for the management of SLE patients, because the presence of atherosclerotic plaques detected by MDCT and myocardial perfusion defects detected by SPECT are strong predictors of death in other populations of patients [28,29,33-35]. Possible beneficial effects of statin treatment on prognosis of SLE patients should, however, be addressed in future large prospective clinical trials.

### Limitations of the study

Although the most commonly used marker of coronary atherosclerosis is calcium scoring, we also measured the volume of calcified plaques in coronary arteries. This is because a major limitation of Agatson calcium score estimation is the measurement of calcium deposits area and density measurement of the calcium (Hounsfield units, HU) itself. The density is assessed using the weighting factor in a stepwise manner, that is not linear or continuous: for calcium measures 130 to 200 HU the density score is one, for 200 to 300 HU the density score is two, etc. [21]. Therefore, small HU difference may yield a major Agatson score difference. Also, its reproducibility is limited to ± 15 to 20%.

Although coronary calcified plaques are proved to be responsible for myocardial ischemia and myocardial infarction, the other mechanisms of coronary flow abnormalities in SLE population should also be underlined. Endothelial damage and/or microthrombosis in coronary bed related to antiphospholipid autoantibodies [36,43,44] was discussed above.

## Conclusions

The SPECT study showed myocardial perfusion defects in 50% of SLE patients despite normal ECG recordings and lack of clinical symptoms of myocardial ischemia. In addition, 25% of patients showed atherosclerotic plaques in coronary arteries.

Treatment with atorvastatin lead not only to the decrease of serum lipids and CRP levels, but also to the limitation of atherosclerosis progression as assessed by MDCT-based calcium scoring. The definite value of statin therapy in SLE patients free of clinical symptoms of cardiovascular disease should be addressed in large prospective clinical trials.

## Abbreviations

aCL: anticardiolipin antibodies; ANA: antinuclear antibodies; ALT: alanine aminotransferase; APS: antiphospholipid syndrome; AST: aspartate aminotransferase; CPK: creatine phosphokinase; CRP: C-reactive protein; ELISA: enzyme linked immunosorbent assay; LA: lupus anticoagulant; LDL: low-density lipoprotein; MDCT: multi-detector computed tomography; SLE: systemic lupus erythematosus; SLEDAI: Systemic Lupus Erythematosus Disease Activity Index; SPECT: single photon emission computed tomography.

## Competing interests

The authors declare that they have no competing interests.

## Authors' contributions

WP was responsible for the study concept and design, acquisition, analysis and interpretation of the data, and manuscript preparation. KG, HD, LTP, and MK acquired and analyzed the data. PP and JM were responsible for data interpretation and manuscript preparation. All authors read and approved the final version of the manuscript.
